# Mid- to long-wave infrared computational spectroscopy using a subwavelength coaxial aperture array

**DOI:** 10.1038/s41598-019-49593-0

**Published:** 2019-09-19

**Authors:** Benjamin J. Craig, Jiajun Meng, Vivek Raj Shrestha, Jasper J. Cadusch, Kenneth B. Crozier

**Affiliations:** 1 School of Physics, University of Melbourne, Victoria, 3010 Australia; 2 Department of Electrical and Electronic Engineering, University of Melbourne, Victoria, 3010 Australia

**Keywords:** Metamaterials, Infrared spectroscopy

## Abstract

Miniaturized spectrometers are advantageous for many applications and can be achieved by what we term the filter-array detector-array (FADA) approach. In this method, each element of an optical filter array filters the light that is transmitted to the matching element of a photodetector array. By providing the outputs of the photodetector array and the filter transmission functions to a reconstruction algorithm, the spectrum of the light illuminating the FADA device can be estimated. Here, we experimentally demonstrate an array of 101 band-pass transmission filters that span the mid- to long-wave infrared (6.2 to 14.2 μm). Each filter comprises a sub-wavelength array of coaxial apertures in a gold film. As a proof-of-principle demonstration of the FADA approach, we use a Fourier transform infrared (FTIR) microscope to record the optical power transmitted through each filter. We provide this information, along with the transmission spectra of the filters, to a recursive least squares (RLS) algorithm that estimates the incident spectrum. We reconstruct the spectrum of the infrared light source of our FTIR and the transmission spectra of three polymer-type materials: polyethylene, cellophane and polyvinyl chloride. Reconstructed spectra are in very good agreement with those obtained via direct measurement by our FTIR system.

## Introduction

Spectroscopy in the mid- to long-wave infrared (MWIR-LWIR) is an important analytical tool, largely because many chemicals have distinct features, such as absorption lines, in these spectral ranges. Example applications of MWIR-LWIR spectroscopy include non-invasive disease diagnosis^1,2^, chemical identification in forensics^3,4^ and food quality testing^5,6^. For many of these applications, it would be very useful to be able to perform spectroscopy in the field rather than being limited to the laboratory setting. This motivates the realization of MWIR-LWIR spectrometers that are much smaller than the traditional platform, i.e. the Fourier transform infrared (FITR) spectrometer. While such spectrometers would not be likely to achieve the resolution of a traditional FTIR spectrometer, the goal would be for them to offer resolution that is sufficient to distinguish between different materials, e.g. enabling absorption lines to be recorded.

One approach to miniaturize spectrometers involves pairing a filter array with a detector array. In these ‘filter-array detector array’ (FADA) devices, the filter array acts as a spectral discriminator. This allows for highly miniaturized devices as the filter arrays can have areas less than 10 *mm*^2^. It has been shown that the spectrum of light illuminating such a device can be determined by reconstruction algorithms^7,8^. These algorithms take as inputs the signals (e.g. photocurrents) measured from the detectors in the array and the transmission spectra of the filters.

FADA spectrometers have been demonstrated using a variety of approaches^7–10^. These devices have integrated photodetector arrays (e.g. charge coupled device image sensors) with a variety of filter arrays. The filters have included quantum dots^7^, Fabry-Perot etalons^8^ and multilayer photonic crystals^9^. While excellent performance has been shown using such filters, their fabrication can be complicated and expensive, with additional steps needed if the number of filters in the array is to be increased.

Plasmonic metasurfaces are an alternative for the filter array. These can be band-stop (e.g. plasmonic nanoantennas) or band-pass (e.g. coaxial apertures of this work) filters, whose spectral responses are determined by the shape, dimensions and material^11^ of the plasmonic nanostructures. The fact that these are patterned from a single metal layer (e.g. gold, silver, aluminum) and that markedly different spectral responses are possible without changing the thickness of this layer is advantageous for manufacturing. This is because the spectral responses of all filters in an array are defined by a single lithography step. It is therefore possible to increase the number of filters in an array without adding steps to the fabrication process. Plasmonic metasurfaces have been used as the basis for filter arrays at visible, NIR and MIR wavelengths for imaging and spectroscopy^12–16^.

Spectroscopy using a plasmonic based FADA device has been demonstrated at NIR wavelengths^12^. At the time of writing, proof-of-concept models based on plasmonics have been demonstrated for spectral reconstruction in the MWIR-LWIR^15,16^. In these works, detector arrays were not used. Rather, in both works the optical power transmitted through each filter of the array was measured using an FTIR microscope, to emulate what would be measured by a detector of a detector array. These works have shown that MWIR-LWIR spectral reconstruction is possible. However, they have failed to show high-resolution reconstruction of important features such as absorption lines. The ability to perform high resolution reconstruction would be important for many potential applications of the FADA approach.

**Figure 1:**
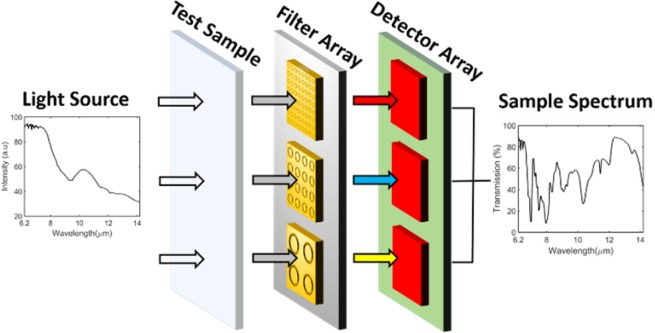
Miniaturized ‘filter-array detector-array (FADA)’ spectrometer model.

Here, we perform a proof-of-principle demonstration of the FADA approach, showing that high resolution reconstruction is possible with plasmonic filters in the MWIR-LWIR spectral range. We fabricate an array of 101 plasmonic metasurface filters, each consisting of a square array of coaxial apertures in a gold film. These function as band-pass filters, with resonances spanning the wavelength range 6.2 *μm*−14.2 *μm*. Our filter array would ideally be used with a detector array to realize a FADA platform for measuring the spectra of test samples, as illustrated in Fig. 1. However, at the time of writing we do not have a suitable detector array and so instead measure the power transmitted through each filter with an FTIR microscope.

The organization of this paper is as follows. In the next section, entitled “Coaxial aperture filter”, we describe the design, simulation, fabrication and optical characterization of plasmonic metasurface filters (coaxial apertures). In “Recursive least squares (RLS) method”, we describe the algorithm^17^ that we use to reconstruct spectra. This algorithm takes the optical power measured through each filter and the filter transmission functions as its inputs. In the section entitled “Results”, we describe our experimental findings. We reconstruct the spectrum of the infrared light source of our FTIR (silicon carbide globar) and the transmission spectra of three polymer materials: polyethylene, cellophane and polyvinyl chloride. We show that these are in very good agreement with the spectra measured directly by the FTIR. In “Conclusion”, we finish this paper with a summary and suggestions for future work.

## Coaxial Aperture Filter

**Figure 2:**
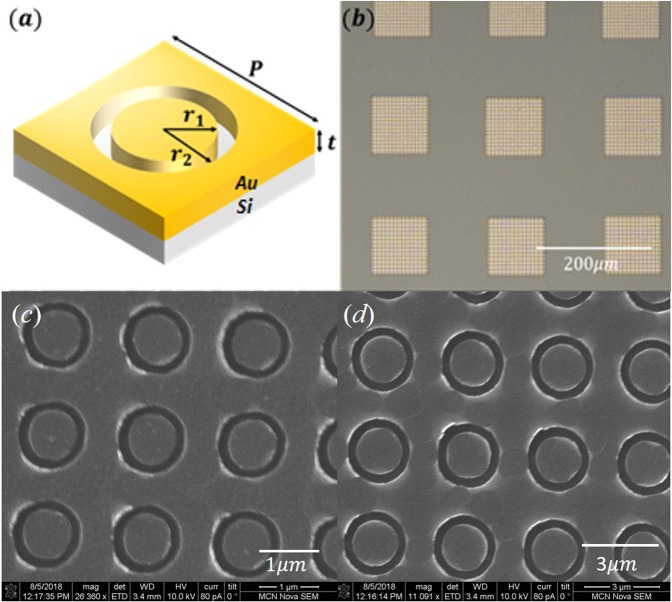
(**a**) Schematic of unit cell of filter, consisting of coaxial aperture. (**b**) Optical microscope image of filters. SEM images of filters with (**c**) P =  1.7 µm and (**d**) P = 3.7 μm.

As discussed, our plasmonic metasurface MWIR-LWIR filters consist of coaxial apertures in a gold film. Coaxial aperture filters have been demonstrated at visible, NIR and MWIR wavelengths^18–22^. In each of our filters, the coaxial apertures (annular vacancies) are arranged in a square array, with a schematic of the unit cell shown as Fig. 2a. The geometric parameters (*r*_1_, *r*_2_, *P*, *t*) determine the resonances supported by the coaxial apertures and therefore their transmission spectra. In this work we vary these parameters to achieve resonances that span the MWIR-LWIR spectral range.

It is seen in the SEM images that the apertures are not completely symmetric. It is also evident that the aperture side walls do not meet the top Au surface at a perfect right angle. This is revealed by the fact that the circumference of each aperture shows inconsistent brightness in the SEM images. These imperfections in the fabrication process do not prevent the filters from being useful for spectral reconstruction. In addition, it can be seen that the dimensions of the fabricated coaxial aperture are in good agreement with design values.

**Figure 3:**
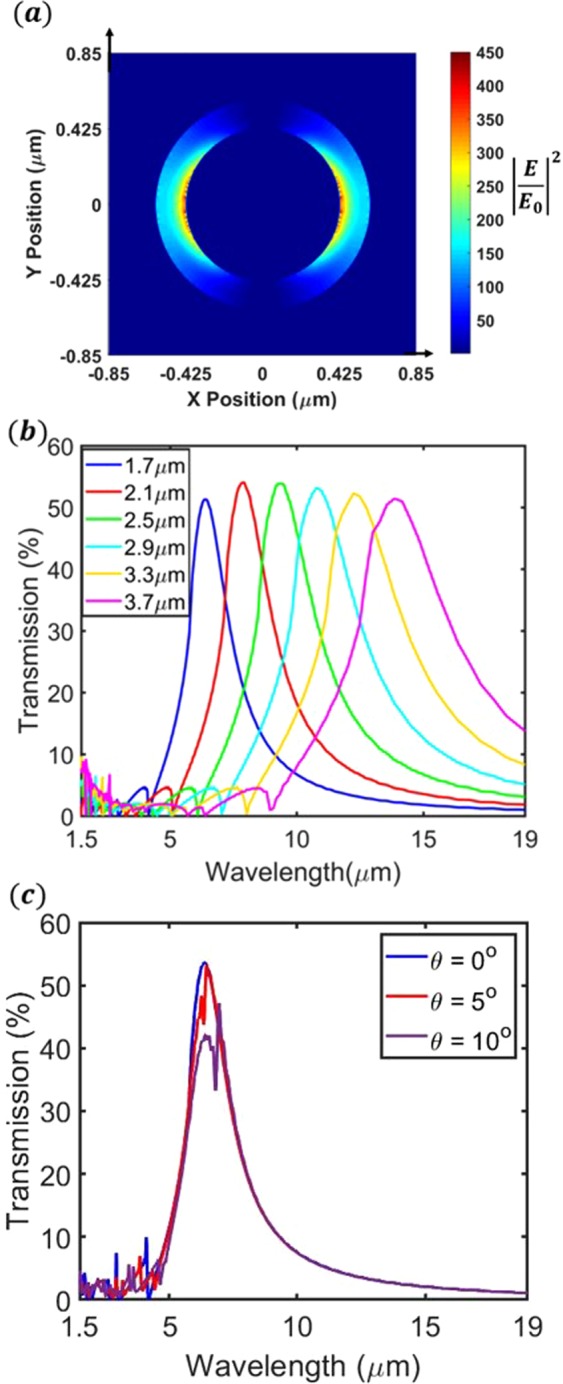
(**a)** Simulated intensity enhancement of coaxial aperture (P = 1.7 µm). (**b**) Simulated transmission spectra of filters with periods P ranging from 1.7 µm–3.7 µm. (**c**) Simulated transmission of filter (P = 1.7 µm) at different incident angles.

It has been theoretically predicted by Baida *et al*.^23^ and Haftel *et al*.^24^ that the extraordinary optical transmission (EOT) of such filters is due to surface plasmons. This has been experimentally confirmed by Orbons *et al*.^20^. Unlike arrays of circular nanoholes (e.g. see^25^), coaxial ring apertures exhibit EOT due to the resonance of each isolated aperture. This is due to the formation of ‘cylindrical’ surface plasmons (CSPs) at each aperture^24^. The simulated local field enhancement resulting for illumination of a device with *P* = 1.7 *μm* at a wavelength of *λ* = 6.4 *μm* is shown as Fig. 3a. In this figure, we plot the intensity enhancement around the coaxial aperture, in response to *x*-polarized plane wave illumination at normal incidence from the Si substrate. Here, *E*_0_ is the amplitude of the electric field of the illuminating plane wave in the Si substrate. The intensity enhancement (Fig. 3a) is plotted at the Si-gold interface (on the gold side).

By varying the geometric parameters of the design (*P*, *r*_1_, *r*_2_, *t*), it possible to vary not only the wavelength of the transmission resonance, but also its full width at half maximum (FWHM), peak transmission value, and the ratio between on- and off-resonance transmission. In this work, we fix the value of *t* (=140 nm), the ratio *r*_1_/*P* (=0.25) and the ratio *r*_2_/*P* (=0.35). Varying the square array period *P* then results in the transmission resonant peak shifting as desired, as confirmed by the finite difference time domain (FDTD) simulations presented as Fig. 3b. In these simulations, the illumination is from the Si substrate side. The transmission plotted in Fig. 3b is the power transmitted into air divided by the power incident from within the Si substrate. It can be seen that each spectrum has a resonance with peak transmission ~50%. In the design of these filters, there is a trade-off between FWHM and peak transmission. As we present later, in this work we demonstrate that the design we implement allows us to achieve spectral reconstructions that are in good agreement with spectra measured directly by our FTIR system. We note that if we were to change the hardware used in the experiment (e.g. source or detector) or the application (e.g. sample), then the optimal geometric parameters of the design would change. For example, if we were to use a detector with lower signal-to-noise ratio, then to maximize the accuracy of spectral reconstruction, it may be appropriate to employ a design with a larger peak transmission value but also with larger FWHM.

Coaxial apertures have advantages over other forms of plasmonic filters, with two examples as follows. First, as they are formed in metal films, they have relatively high on-resonance transmission and relatively low off-resonance transmission. In other words, they function well as pass-band filters. By comparison, stop-band filters based on plasmonic antennas often have appreciable transmission in their stop-bands. Second, the resonant wavelength is relatively insensitive to the angle of illumination. Figure 3c shows the simulated transmission of a filter with *P* = 1.7 *μm* at three illumination angles (*θ*), with the incident wave being aligned along a major axis of the array (e.g. *ϕ* = 0° in standard spherical coordinates). As before, illumination is from within the Si substrate and the plotted transmission represents the power transmitted into air divided by the power incident from within the Si. For the two spectra at non-normal incidence (*θ* = 5°, 10°), transmission spectra for s- and p-polarizations are simulated and averaged. Note that these angles refer to the angle of incidence within the Si substrate. The simulations show that the spectral position of the zeroth-order resonance does not change appreciably, though its peak transmission decreases with increasing angle. This characteristic is an advantage over conventional cylindrical hole filters, whose resonances are strongly angularly sensitive (e.g. see^25^).

Each filter is a square array of coaxial apertures in an Au layer (140 nm thick, with 2 nm of Cr for adhesion) on a double-side polished undoped silicon substrate, with an overall extent of ~100 *μm* × 100 *μm*. The period (*P*) of the coaxial aperture unit cell varies from 1.7 *μm* to 3.7 *μm* in increments of 20 nm, meaning that there are 101 filters. The inner (*r*_1_) and outer radii (*r*_2_) are set to be 0.25 × *P* and 1.4 × *r*_1_ respectively. Fabrication starts with the spin coating of resist (PMMA A-6, 400 nm thick) onto the substrate (double-side polished undoped silicon wafer). The resist is patterned by electron-beam lithography (Vistec EBPG5000plusES) at a dose of 755 *μC*/*cm*^2^. After development, Cr (2 nm) and then Au (140 nm) films are deposited via electron-beam evaporation (Intlvac Nanochrome II). Lift-off is then performed (hot acetone, with sonication). The sample is then cleaned with acetone and isopropyl alcohol and blown dry with nitrogen.

**Figure 4:**
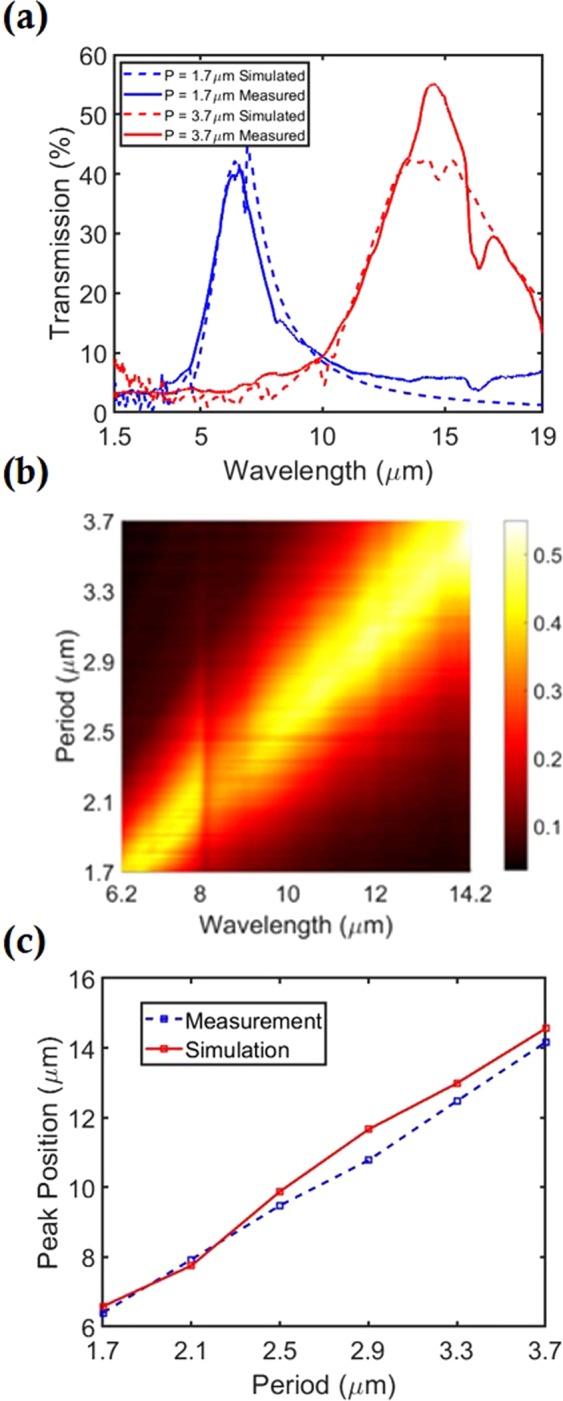
(**a**) Comparison between simulated and measured absolute transmission spectra for coaxial apertures with P = 1.7 µm and P = 3.7 µm. Resonance position and peak transmission are in good agreement. (**b**) Measured transmission spectra for all 101 filters. (**c**) Simulated and measured peak positions vs period.

The transmission function of each filter is measured using an FTIR microscope. From Fig. 4a, it is seen that the measured and simulated spectra are in good agreement (for filters with *P* = 1.7 *μm* and *P* = 3.7 *μm*), both in resonance position and peak transmission. In our simulations, illumination of the filter is from the Si substrate, at an angle of 10°. This is done for the following reason. The reflecting objective of the FTIR microscope has a numerical aperture of *NA* = 0.6, corresponding to illumination angles up to ~37°. The filters are illuminated through the underside of the Si substrate (*n*~3.4). Therefore, the light transmitted into the Si substrate is incident on the Si-air interface at angles up to ~10°. Some discrepancies between simulated and measured transmission may be due to the assumption of 10° illumination in the simulations. In experiments, light from the reflecting objective is spread over a range of illumination angles. As seen in Fig. 3c, smaller angle contributions from the reflecting objective would result in higher peak transmission. Fabrication imperfections may also contribute to the discrepancies.

In the simulations of Fig. 4, to allow comparison with experiment, the effect of multiple internal reflections within the substrate are taken into consideration. It should be noted that this differs from Fig. 3. The simulated total transmission is calculated using:

1 $${T}_{total}={T}_{1}{T}_{2}(1+{R}_{1}{R}_{2}+{R}_{1}^{2}{R}_{2}^{2}+\ldots )$$

where *T*_1_ and *R*_1_ the transmission and reflection at the air-Si interface respectively (calculated using the Fresnel equations and averaged between s- and p-polarizations), *T*_2_ and *R*_2_ are the simulated transmission and reflection at the Si-air interface, respectively (averaged between s- and p-polarizations). It should be noted that, at this interface, the silicon is covered with a gold film and that the coaxial apertures are formed in this film. The thickness of the substrate is orders of magnitude greater than the effective wavelengths being considered. It is therefore reasonable to assume that the transmission can be predicted by summation of the reflected intensities, rather than the electric fields.

The measured transmission spectra of all filters are provided as Fig. 4b. Each spectrum comprises 2801 data points, with a separation of ~3 *nm* between successive points. The peak position shifts approximately linearly from 6.2 *μm*−14.2 *μm* and the filter FWHM increases for increasing period. For all spectra, a transmission dip feature occurs at ~8.1 *μm*. This may be due to absorption by impurities (e.g. resist). It can also be seen that there is a feature at *P* = 2.5 *μm*. This is because the filter with this period *P* = 2.5 *μm* has fabrication imperfections that modify its transmission spectrum slightly. The nature of the plotting method employed in Fig. 4b (colorbar) make this difference very apparent. It can be seen from Fig. 4c that the transmission resonance positions of the measured filters are consistent with simulations.

## Recursive Least Squares (RLS) Method

If the FADA approach is implemented using arrays of band-pass filters with narrow passbands, then spectral reconstruction is relatively straightforward. Indeed, direct readout of the photodetector signals would give a reasonable approximation in that case. In this work, however, we use band-pass filters with passbands that overlap. More sophisticated computational methods are therefore needed for reconstruction. Computational spectroscopy is also applicable to systems containing photodetectors with tailored responsivity spectra such as nanowires (e.g.^26,27^). We use the recursive least squares (RLS) method. This approach recursively determines coefficients that minimize a weighted quadratic least cost function related to the system inputs. The inputs are the transmission functions of the array (*F*_2801×101_) and the signals transmitted through the array (*S*_1×101_). The minimization coefficients determined are the incident spectrum. For the *i*^*th*^ recursive step, the *i*^*th*^ filter’s transmission (i.e. *i*^*th*^ column of *F*_2801×101_, *F*_*i*_) and signal data (i.e. *i*^*th*^ entry of *S*_1×101_, *S*_*i*_) are input into the algorithm. The minimization coefficients are then recalculated. The recursive method is repeated until all data (i.e. *F*_2801×101_ and *S*_1×101_) has been input to the algorithm. The final minimization coefficients are the algorithm’s estimate of the incident spectrum. A full derivation of the governing equations of the RLS method is not provided here as this can be found from standard texts such as ref.^17^. The weighted least squares cost function (*C*) we desire to minimize is:

2 $$C=\mathop{\sum }\limits_{i=1}^{n}\,{\delta }^{n-i}{e}_{i}^{2}$$

where *n* is the number of steps (=101), *e*_*i*_ is the difference between the signal data of the *i*^*th*^ filter and product of the *i*^*th*^ filter’s transmission and the recursive solution *X*_*i*_ at the *i*^*th*^ step, i.e:

3 $${e}_{i}={F}_{i}^{T}{X}_{i}-{S}_{i}$$

The recursive solution (*X*_*i*_) for the minimization of the cost function at the *i*^*th*^ step is:

4 $${X}_{i}={X}_{i-1}+{P}_{i}{F}_{i}({S}_{i}-{F}_{i}^{T}{X}_{i-1})$$

where

5 $${P}_{i}=[{P}_{i-1}-{P}_{i-1}{F}_{i}{(\delta I+{F}_{i}^{T}{P}_{i-1}{F}_{i})}^{-1}{F}_{i}^{T}{P}_{i-1}]{\delta }^{-1}$$

where *F*_*i*_^*T*^ is the row vector form of the *i*^*th*^ filter vector *F*_*i*_, *P*_*i*_ is the RLS covariance matrix, *I* is the identity matrix and *δ* is the ‘forget factor’ (0 < *δ* ≤ 1). *P* is initialized as a matrix (2801 × 101) with ones along the diagonal and zeroes elsewhere. The spectrum *X* is initialized to be a null vector, i.e. each entry is zero. *δ* determines the weighting given to previously inputted filter data. Here, we input filter data to the algorithm in order of largest to smallest period filters. This is done because the filter FWHM decreases as the period decreases. Providing the data in this way means that the data from the filters with narrower FWHM is input later and thus prioritized. This allows the algorithm to correct for errors that emerge due to the overlapping and broad nature of the transmission peaks. For this work, *δ* is kept constant at 0.96 for all reconstructions. It can be seen from the next section that this choice leads to accurate reconstructions.

## Results

**Figure 5:**
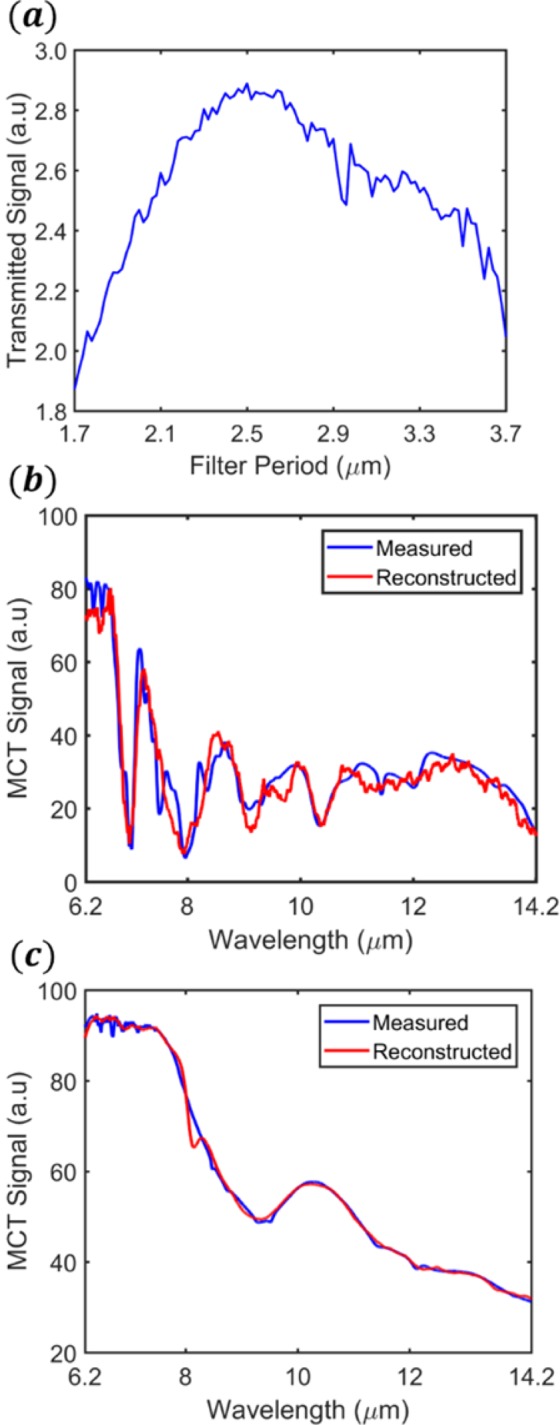
(**a**) Integrated transmitted signal of each filter (S-vector) for the cellophane test sample. Reconstructions of (**b**) cellophane ((i.e. X^M^ (*λ*), NMAE = 0.0446) and (**c**) globar (i.e. X^I^ (*λ*), NMAE = 0.0071).

Next, we demonstrate spectral reconstruction using the filter array. To demonstrate our approach, we perform spectral reconstructions to determine the transmission spectra of three polymer-based materials: cellophane, polyvinyl chloride and polyethylene. Ideally, the filter array would be combined with a detector array. As discussed, at the time of writing, we do not have an appropriate detector available to us, so instead we measure the light transmitted through each filter with our FTIR microscope, to emulate what would be measured by each detector of a detector array. The spectral reconstruction is performed as follows. The transmission spectrum of each filter *F*_*i*_(*λ*) is measured by the FTIR microscope, representing a column of the filter matrix *F*_2801×101_ (Fig. 4b). Next the material being tested is placed in the infrared beam path of our FTIR system. For convenience, we choose to place the material at the location where the infrared beam passes from the FTIR unit (Perkin Elmer Frontier) to the FTIR microscope (Perkin Elmer Spotlight). The total optical signal *S*_*i*_^*M*^ transmitted through each filter is then determined by measuring the spectrum transmitted through each filter. The time taken by the FTIR system for the measurement of each spectrum is on the order of seconds. We then integrate over the wavelength range of interest (6.2 *μm* to 14.2 *μm*). This represents,

6 $${S}_{i}^{M}=\int I(\lambda )\times M(\lambda )\times MC{T}_{resp}(\lambda )\times {F}_{i}(\lambda )d\lambda $$

where *I*(*λ*) is the intensity spectrum of the infrared source of our FTIR (silicon carbide globar), *M*(*λ*) is the transmission spectrum of the material being tested, and *MCT*_*resp*_(*λ*) is the responsivity of the mercury cadmium telluride (MCT) detector of our FTIR microscope. The transmitted signals form the vector ${S}_{1\times 101}^{M}$. This vector emulates what would be measured by a detector array. Figure 5a shows ${S}_{1\times 101}^{M}$ resulting from measurements of the cellophane test sample.

The filter matrix *F*_2801×101_ and the transmitted signal vector ${S}_{1\times 101}^{M}$ are then input into the RLS algorithm. The output of the RLS algorithm is (ideally) the following spectrum:

7 $${X}^{M}(\lambda )=I(\lambda )\times M(\lambda )\times MC{T}_{resp}(\lambda )$$

The reconstructed intensity spectrum *X*^*M*^(*λ*) for cellophane is shown in Fig. 5b. We next reconstruct the transmission function *M*(*λ*) of the test material. To do so, we need to reconstruct the spectrum measured with no test material present, which we refer to as the reconstructed globar spectrum *X*^*I*^(*λ*). This is (ideally) given by:

8 $${X}^{I}(\lambda )=I(\lambda )\times MC{T}_{resp}(\lambda )$$

We then reconstruct the material transmission spectrum by dividing *X*^*M*^(*λ*) by the reconstructed globar spectrum:

9 $$M(\lambda )={X}^{M}(\lambda )/{X}^{I}(\lambda )$$

The reconstructed globar intensity spectrum *X*^*I*^(*λ*) is shown in Fig. 5c. The accuracy of the reconstructions is quantified by the normalized mean-absolute-error (NMAE). This is the mean of the absolute difference between the spectrum measured by the FTIR (*Y*(*λ*)) and that reconstructed by our system, normalised to the peak signal value (*m*, from spectrum measured by FTIR), i.e:

10 $$NMAE=\frac{{\sum }_{\,}^{\lambda \,}\,|Y(\lambda )-\,X(\lambda )|}{k\times m}$$

Here, *k* denotes the number of points in each spectrum (=2801), i.e. the number of terms in the summation of Eq. (10). It can be seen that the reconstructions of cellophane (*X*^*M*^(*λ*), Fig. 5b) and the globar (*X*^*M*^(*λ*), Fig. 5c) spectra are in good agreement with spectra measured directly by the FTIR, as evidenced by low NMAE values (0.0446 and 0.0071, respectively).

In this work several reconstructions are performed for each material, using *S*-vectors that are slightly different because they are the result of measurements performed at different times. We next discuss how we choose the reconstruction that is expected to be the most accurate. It is of course important that our method for choosing does not require any a-priori knowledge of the spectrum being reconstructed. Our method compares the measured *S*-vector (*S*^*M*^) with the *S*-vector (*S*^*R*^) that would result if the reconstructed spectrum were the actual spectrum, i.e.

11 $$\,{S}_{1\times 101}^{R}={X}_{1\times 2801}^{M}\times {F}_{2801\times 101}$$

Next, we calculate,

12 $$\Delta {S}_{i}=(|{S}_{i}^{R}-{S}_{i}^{M}|\times 100)/{S}_{i}^{M}$$

**Figure 6:**
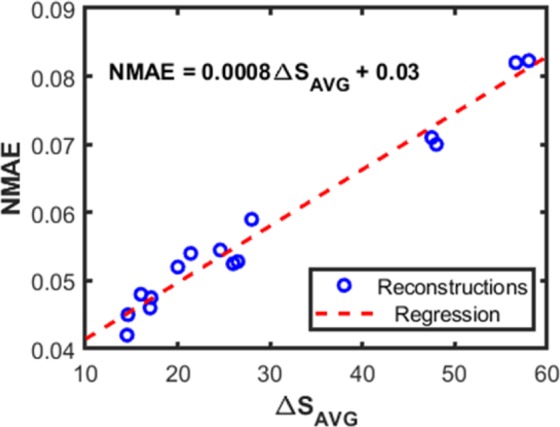
A set of polyvinyl chloride reconstruction. Linear regression shows high correlation between NMAE and ∆S_Avg_, with R^2^ = 0.968 and p-value of 4.64e-11.

This defines the absolute error, as a percentage, of *S*^*R*^ for each filter, when compared to *S*^***M***^. Note that in Eq. (12), the division is performed in an element-wise fashion. Δ*S*_1×101_ is then averaged over all filters, giving Δ*S*_*Avg*_. We then choose the reconstruction with the lowest Δ*S*_*Avg*_. As discussed, this method requires no a-priori knowledge of the spectrum being reconstructed. To quantify the accuracy of this method, in Fig. 6 we plot NMAE against Δ*S*_*Avg*_ for 15 reconstructions performed for polyvinyl chloride. This figure clearly illustrates the trend that NMAE decreases as Δ*S*_*Avg*_ decreases. The polyvinyl chloride *X*^*M*^(*λ*) selected is the one that gives the lowest value for Δ*S*_*Avg*_. This method is repeated for the other materials to select the best reconstruction. The numbers of reconstructions performed for polyvinyl chloride, cellophane and polyethylene are 15, 20 and 20 respectively. For the globar source, six reconstructions are performed. The reconstructions are performed using MATLAB (MathWorks, MA, USA). Each reconstruction takes less than one minute on a desktop computer that can be considered standard at the time of writing (EliteDesk800 from Hewlett Packard).

**Figure 7:**
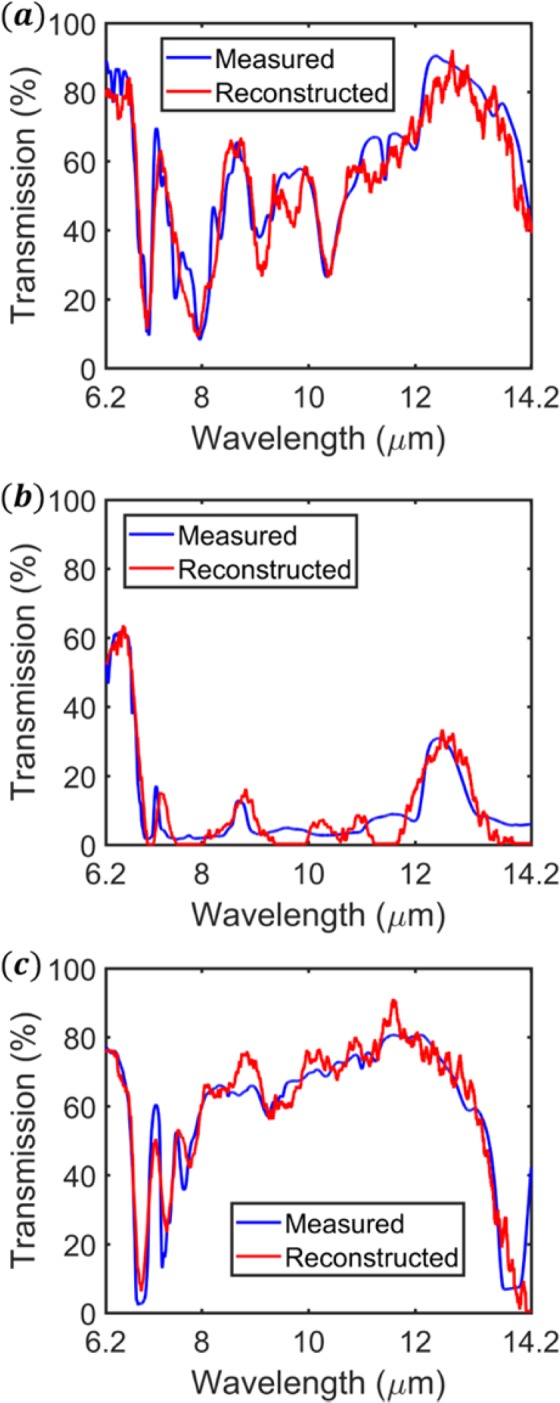
Reconstruction of the material transmission spectra M(*λ*) for (**a**) cellophane (NMAE = 0.0590, ∆S_Avg_ = 0.0582%), (**b**) polyvinyl chloride (NMAE = 0.0605, ∆S_Avg_ = 14.7543%) and (**c**) polyethylene (NMAE = 0.0703, ∆S_Avg_ = 0.0834%).

The reconstructions of *M*(*λ*) for the three materials tested are shown in Fig. 7a–c. The reconstructions are in good agreement with the spectra measured directly by the FTIR. This is evident by the low NMAE value for each material. The reconstruction of cellophane (Fig. 7a) captures many high-resolution features. It is noted that many of the reconstructed features are spectrally narrower than transmission resonances of any of the filters (Fig. 4a,b). Figure 7b clearly shows the sharp “cut-off wavelength” (beyond which transmission is low) of polyvinyl chloride at ~7 *μm* and reproduces the three transmission peaks that occur at longer wavelengths. We note that the spectrum of polyvinyl chloride, as reconstructed by the RLS algorithm, exhibits negative values in several regions. Such values are unphysical and have thus been set to be very small positive values (0.001), with the results shown as Fig. 7b. The practice of setting negative numbers to zero has been applied in other inversion problems^28^. In Fig. 7c we see that the absorption lines of polyethylene (e.g. at *λ*~6.8 *μm*, 7.2 *μm*, 7.7 *μm*) are reproduced well. The first line is almost completely reconstructed, in both depth and spectral width. The remaining features are reproduced with relatively high accuracy. These results far exceed the accuracy shown thus far, i.e. at the time of writing, of previous works on spectral reconstruction with plasmonics in this spectral range^15,16^. In those works^15,16^, the transmission spectrum of polyethylene was also reconstructed. It can be seen that while the multiple absorption lines around *λ*~6.8 *μm* to *λ*~7.7 *μm* are reproduced in the reconstruction of this work (Fig. 7c), the spectral reconstructions of both previous works (Fig. 5d of ^15^ and Fig. 5b of ^16^) each exhibit only one broad dip in that wavelength range.

While the reconstruction spectra are in good agreement with those measured directly by the FTIR, there is room for improvement. Narrow features with small amplitudes are not well reconstructed (e.g. Figure 7a, dip at ~7.6 *μm*). Anomalous features can also appear in the reconstructions (e.g. Figure 7a, dip at ~9.8 *μm*). It is also evident from Fig. 7b that the parts of the spectrum with low transmission away from spectral peaks and dips are not reproduced well. The reconstruction for those parts varies between the desired value and zero. We also observe a high value for ΔS_Avg_ for the reconstruction of polyvinyl chloride (14.7543%). However, this is due to the very low transmission of the material over the wavelength range under study. The low transmission causes most of the elements of ${S}_{1\times 101}^{M}$ to take small values. As such, errors in ${S}_{1\times 101}^{R}$ that are small in an absolute sense will result in ΔS_Avg_ taking a large value.

For all materials, the reconstruction is more error prone at longer wavelengths. We postulate three possible reasons. The first is that the globar signal is smaller at longer wavelengths. It can be seen from Fig. 5c that the combination of the globar spectrum and the MCT detector response at the long wavelength end of the spectrum is only around one third of the value at the short wavelength end. The resultant lower signal-to-noise ratio at long wavelengths could result in increased reconstruction error. The second reason is that the longer-wavelength filters have larger FWHM values, meaning that there is overlap between adjacent filters. As the filter functions become less differentiable, so does their signal data. This leads to reduced accuracy of reconstruction in this region. The third reason is that of the recursive nature of the RLS method. The RLS method sequentially inputs filter data. As we input filter data from largest to smallest period, we prioritize information gained from the filters with shorter wavelength resonances. As the reconstruction evolves, the data with the most relevance to the longer wavelengths becomes less important. This could cause increased error in this region.

These sources of error could be alleviated by refinement of the RLS method. It may also be interesting to consider methods such as Tikhonov regularization^29–31^) and by employing new approaches to achieve band pass filters with smaller FWHM values^32^. Regarding the latter, as discussed earlier, whether a more accurate reconstruction would be obtained using filters with smaller FWHM values would depend on a variety of factors such as detector noise and the nature of the spectrum being measured.

## Conclusion

In this work we demonstrate an array of Au coaxial aperture filters. These are fabricated on a double side polished undoped Si substrate using conventional fabrication techniques. We demonstrate the MWIR-LWIR reconstruction of the transmission spectra of three polymer materials. The complex spectra of these materials are reconstructed well, with accurate reconstruction of the depth and width of absorption lines. Our results represent a substantial advance over the current literature. We demonstrate the efficacy of the method we use to select from multiple reconstructed spectra (e.g. from repeated measurements) the spectrum that is expected to be the most accurate. The future integration of our filters with detector arrays could form the basis for lightweight, portable and inexpensive filter-array detector-array (FADA) spectrometers. These would find a variety of applications, including non-invasive medical diagnosis, forensics and food testing.
